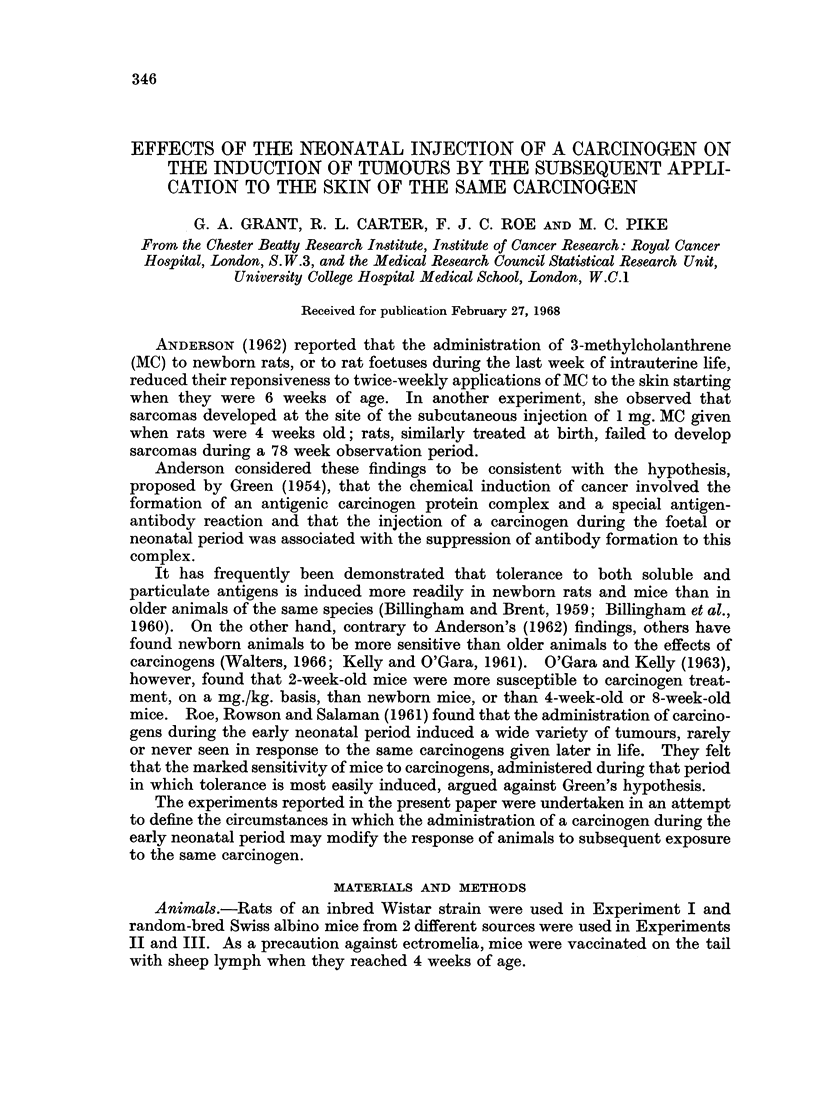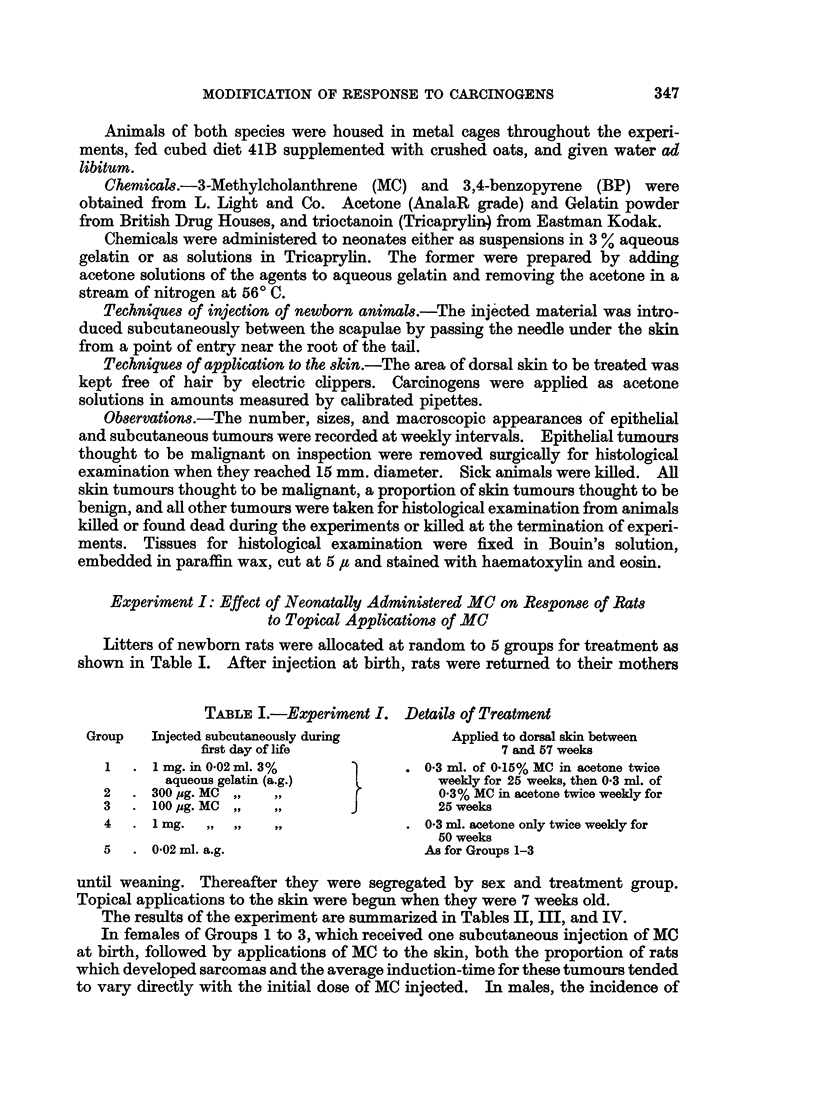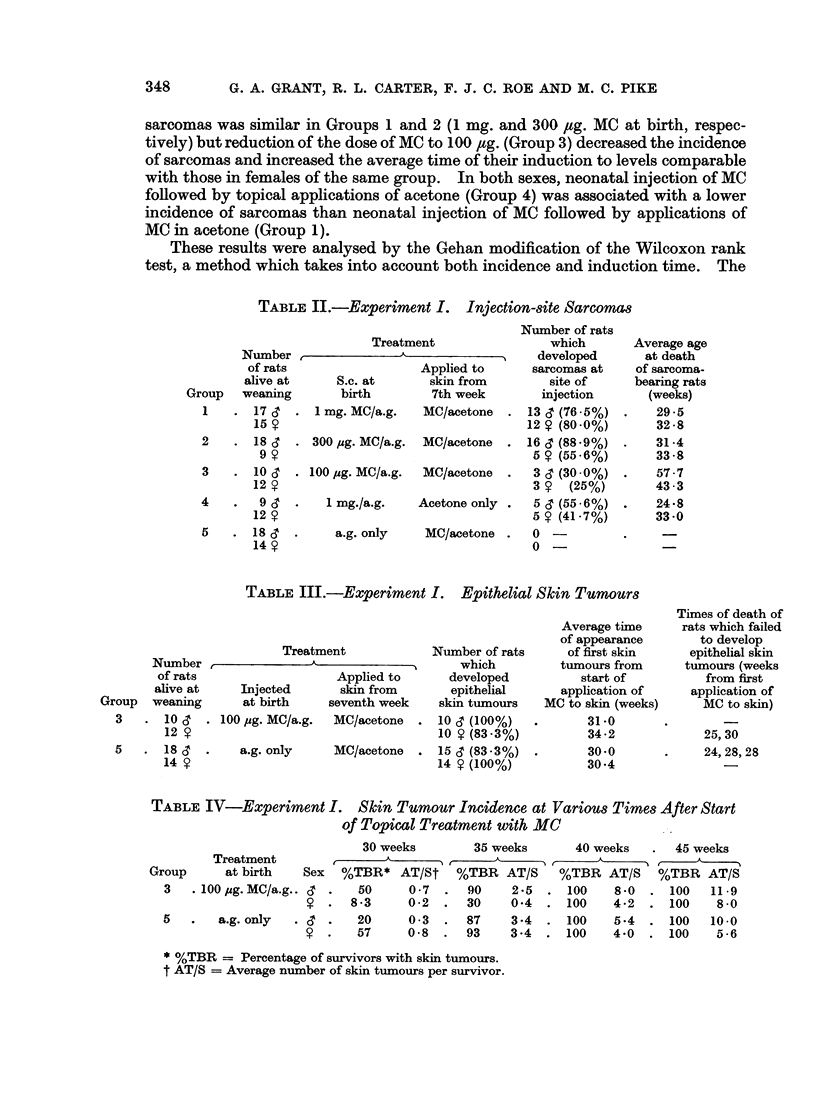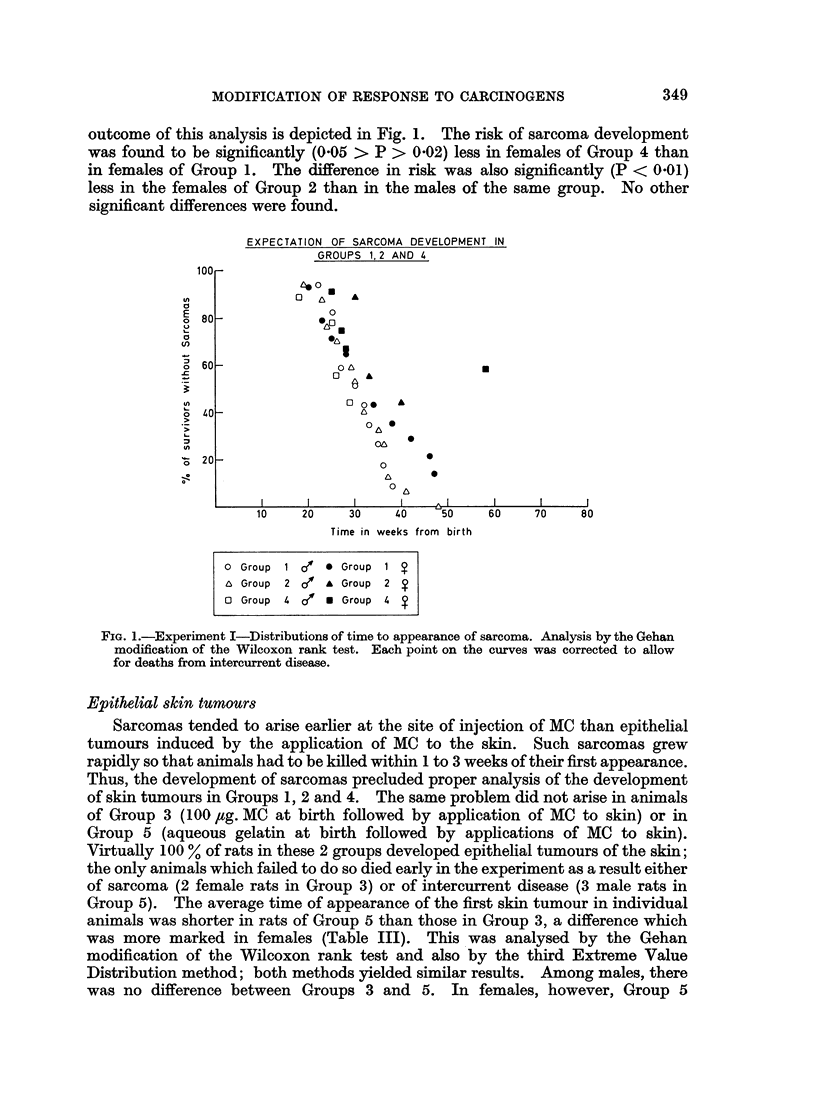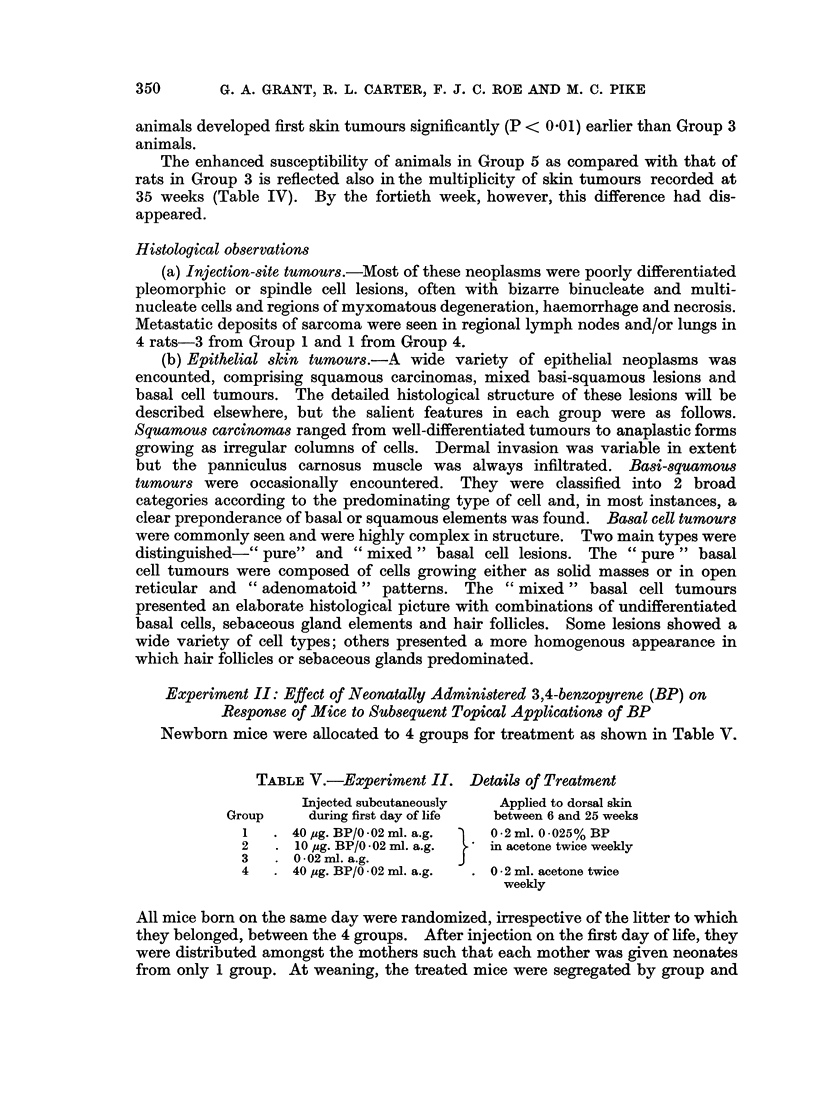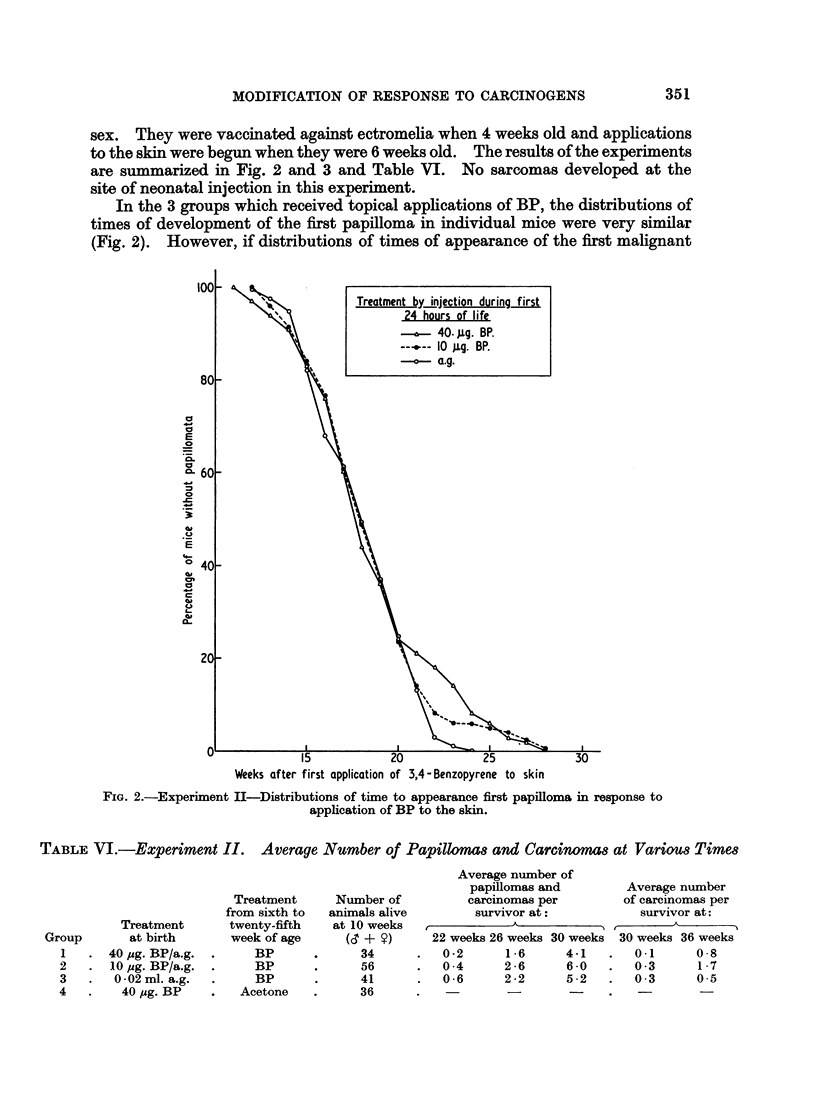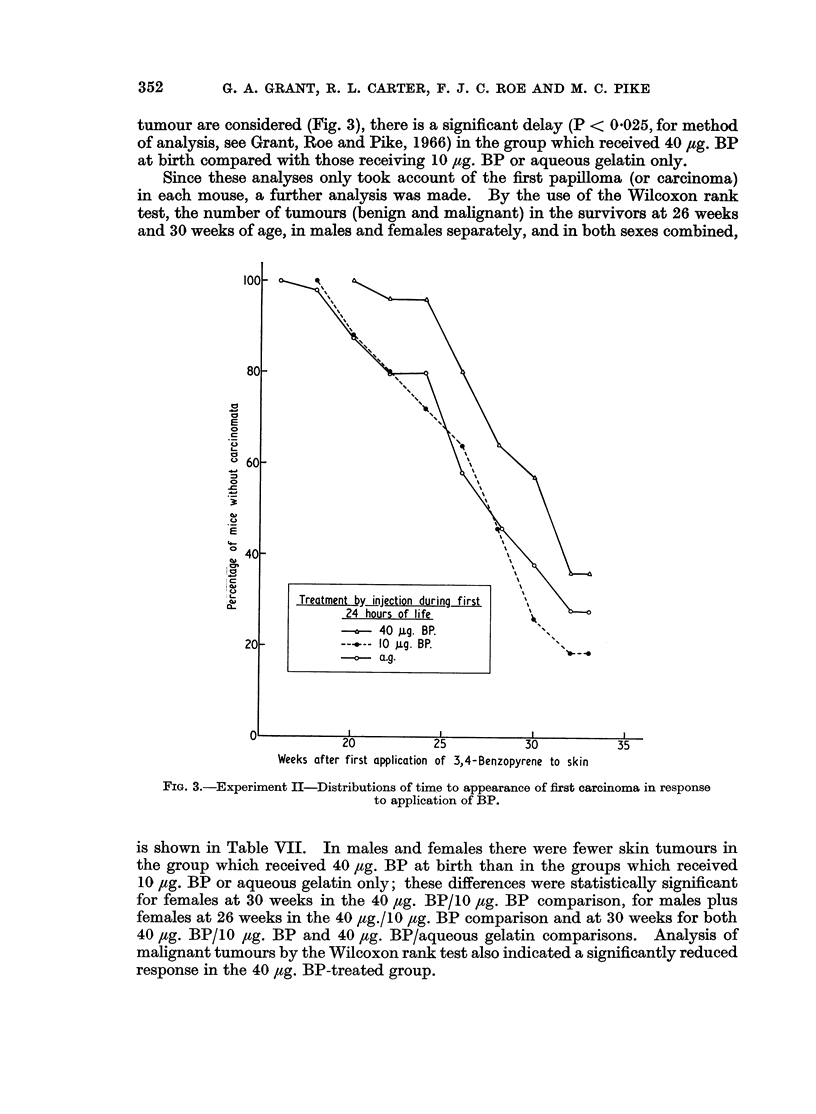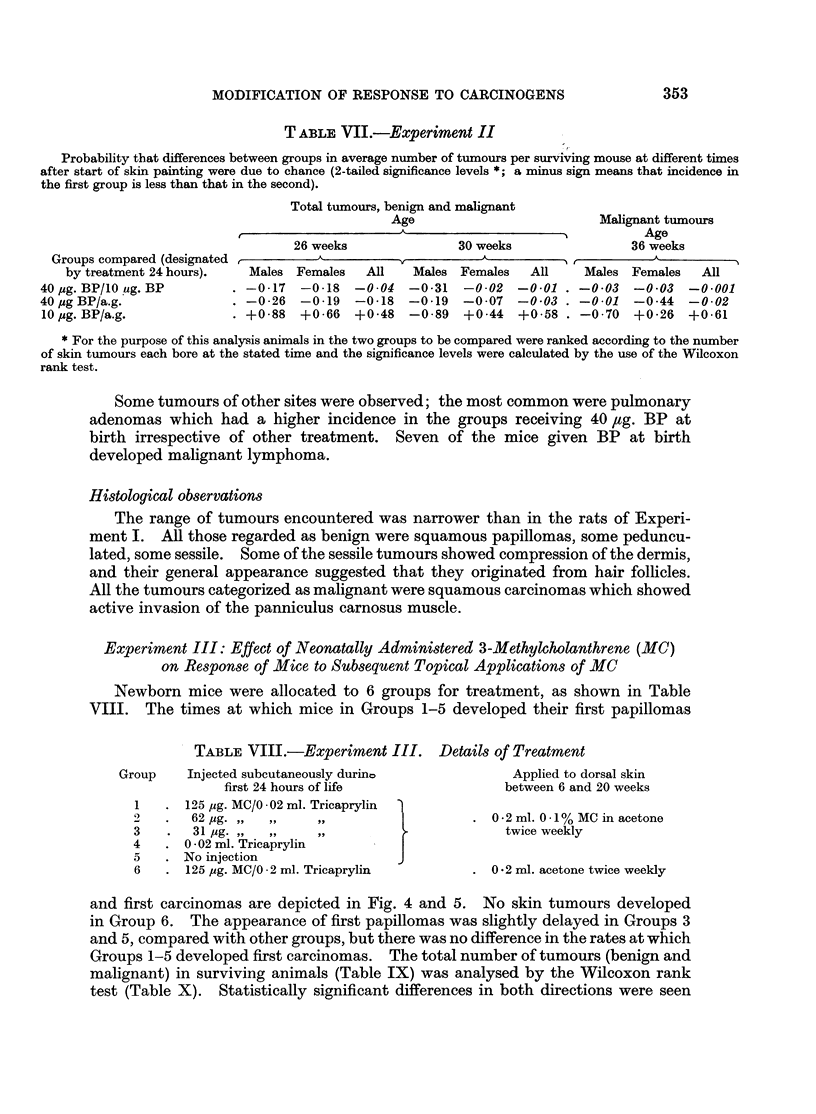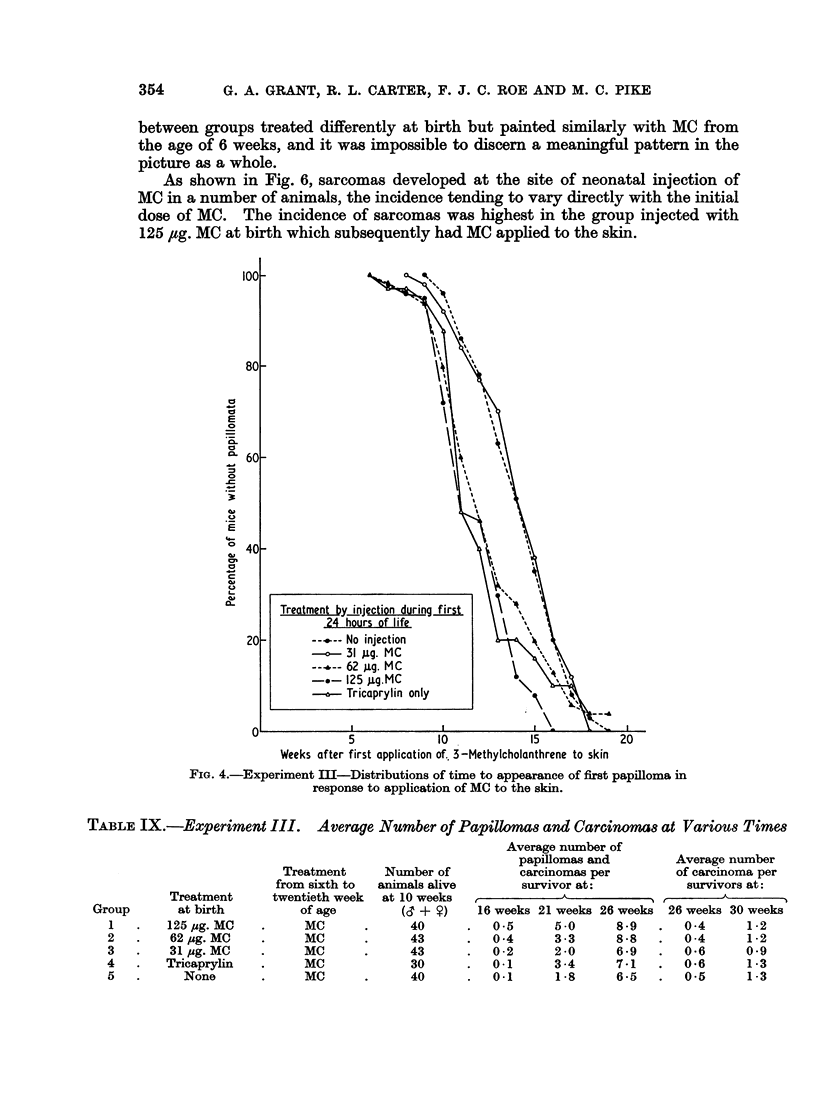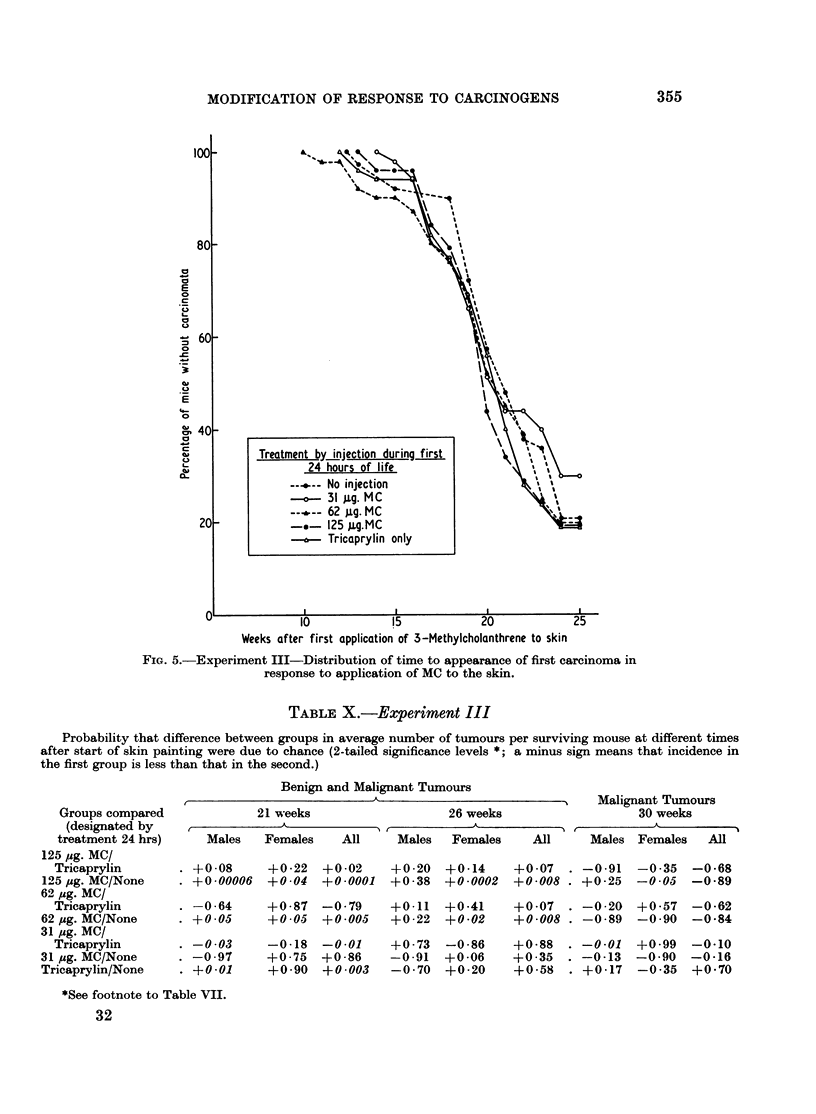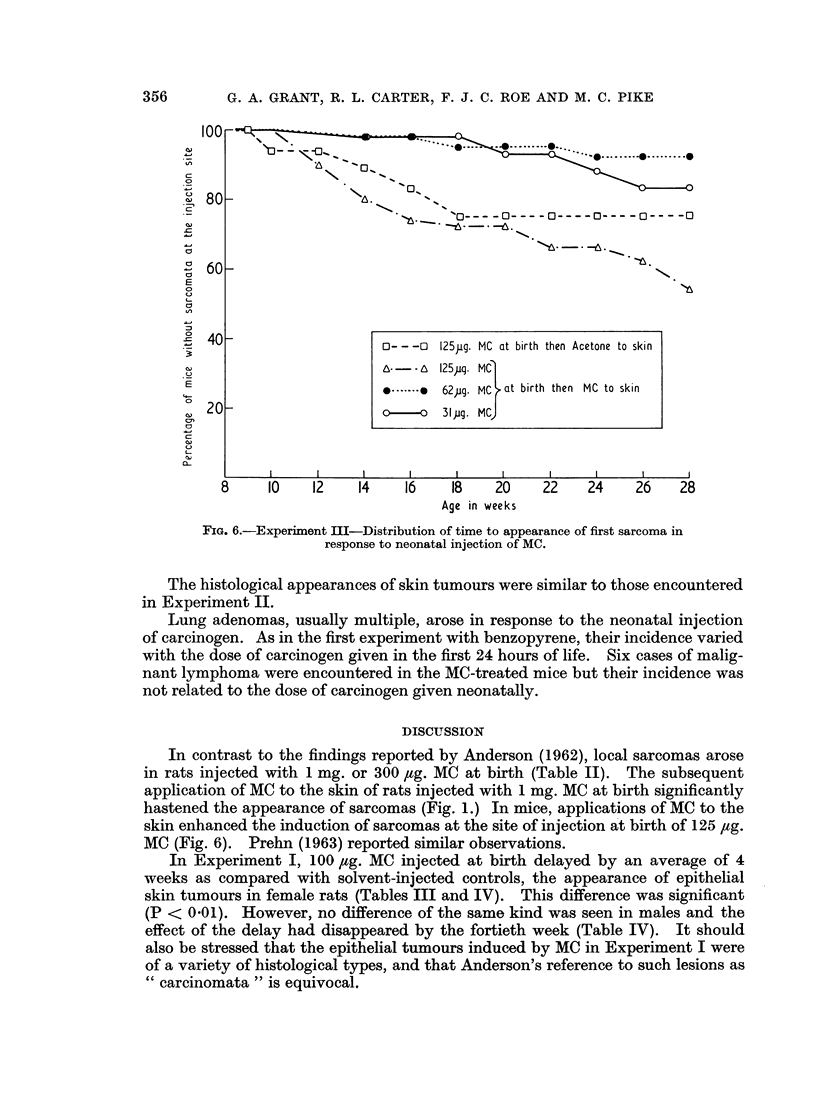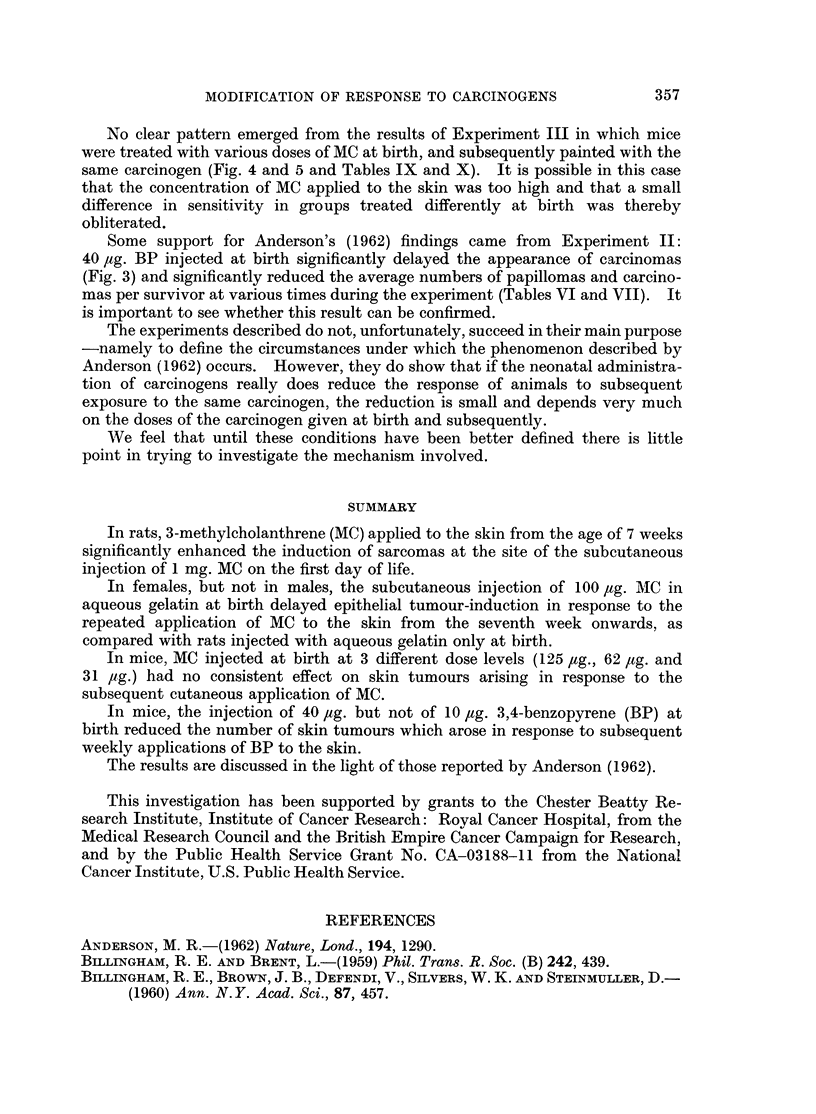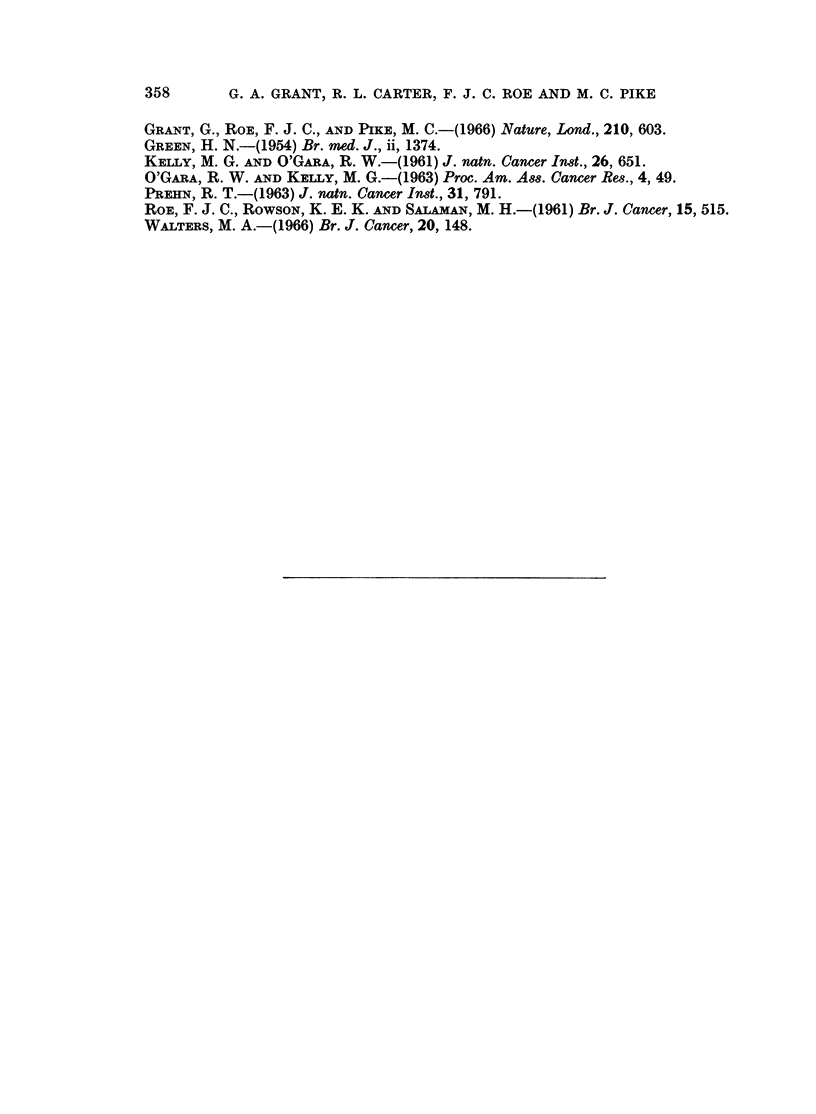# Effects of the neonatal injection of a carcinogen on the induction of tumours by the subsequent application to the skin of the same carcinogen.

**DOI:** 10.1038/bjc.1968.43

**Published:** 1968-06

**Authors:** G. A. Grant, R. L. Carter, F. J. Roe, M. C. Pike


### EFFECTS OF THE NEONATAL INJECTION OF A CARCINOGEN ON THE INDUCTION OF TUMOURS BY THE SUBSEQUENT APPLICATION TO THE SKIN OF THE SAME CARCINOGEN

G. A. GRANT, R. L. CARTER, F. J. C. ROE AND M. C. PIKE

*From the Chester Beatty Research Institute, Institute of Cancer Research: Royal Cancer Hospital, London, S.W.3, and the Medical Research Council Statistical Research Unit, University College Hospital Medical School, London, W.C.1*

Received for publication February 27, 1968

ANDERSON (1962) reported that the administration of 3-methylcholanthrene (MC) to newborn rats, or to rat foetuses during the last week of intrauterine life, reduced their reponsiveness to twice-weekly applications of MC to the skin starting when they were 6 weeks of age. In another experiment, she observed that sarcomas developed at the site of the subcutaneous injection of 1 mg. MC given when rats were 4 weeks old; rats, similarly treated at birth, failed to develop sarcomas during a 78 week observation period.

Anderson considered these findings to be consistent with the hypothesis, proposed by Green (1954), that the chemical induction of cancer involved the formation of an antigenic carcinogen protein complex and a special antigen-antibody reaction and that the injection of a carcinogen during the foetal or neonatal period was associated with the suppression of antibody formation to this complex.

It has frequently been demonstrated that tolerance to both soluble and particulate antigens is induced more readily in newborn rats and mice than in older animals of the same species (Billingham and Brent, 1959; Billingham *et al.*, 1960). On the other hand, contrary to Anderson's (1962) findings, others have found newborn animals to be more sensitive than older animals to the effects of carcinogens (Walters, 1966; Kelly and O'Gara, 1961). O'Gara and Kelly (1963), however, found that 2-week-old mice were more susceptible to carcinogen treatment, on a mg./kg. basis, than newborn mice, or than 4-week-old or 8-week-old mice. Roe, Rowson and Salaman (1961) found that the administration of carcinogens during the early neonatal period induced a wide variety of tumours, rarely or never seen in response to the same carcinogens given later in life. They felt that the marked sensitivity of mice to carcinogens, administered during that period in which tolerance is most easily induced, argued against Green's hypothesis.

The experiments reported in the present paper were undertaken in an attempt to define the circumstances in which the administration of a carcinogen during the early neonatal period may modify the response of animals to subsequent exposure to the same carcinogen.

### MATERIALS AND METHODS

*Animals.*—Rats of an inbred Wistar strain were used in Experiment I and random-bred Swiss albino mice from 2 different sources were used in Experiments II and III. As a precaution against ectromelia, mice were vaccinated on the tail with sheep lymph when they reached 4 weeks of age.

Animals of both species were housed in metal cages throughout the experiments, fed cubed diet 41B supplemented with crushed oats, and given water *ad libitum.*

*Chemicals.*—3-Methylcholanthrene (MC) and 3,4-benzopyrene (BP) were obtained from L. Light and Co. Acetone (AnalaR grade) and Gelatin powder from British Drug Houses, and trioctanoin (Tricaprylin) from Eastman Kodak.

Chemicals were administered to neonates either as suspensions in 3 % aqueous gelatin or as solutions in Tricaprylin. The former were prepared by adding acetone solutions of the agents to aqueous gelatin and removing the acetone in a stream of nitrogen at 56° C.

*Techniques of injection of newborn animals.*—The injected material was introduced subcutaneously between the scapulae by passing the needle under the skin from a point of entry near the root of the tail.

*Techniques of application to the skin.*—The area of dorsal skin to be treated was kept free of hair by electric clippers. Carcinogens were applied as acetone solutions in amounts measured by calibrated pipettes.

*Observations.*—The number, sizes, and macroscopic appearances of epithelial and subcutaneous tumours were recorded at weekly intervals. Epithelial tumours thought to be malignant on inspection were removed surgically for histological examination when they reached 15 mm. diameter. Sick animals were killed. All skin tumours thought to be malignant, a proportion of skin tumours thought to be benign, and all other tumours were taken for histological examination from animals killed or found dead during the experiments or killed at the termination of experiments. Tissues for histological examination were fixed in Bouin's solution, embedded in paraffin wax, cut at 5 μ and stained with haematoxylin and eosin.

### *Experiment I: Effect of Neonatally Administered MC on Response of Rats to Topical Applications of MC*

Litters of newborn rats were allocated at random to 5 groups for treatment as shown in Table I. After injection at birth, rats were returned to their mothers

TABLE I.—*Experiment I. Details of Treatment*

| Group | Injected subcutaneously during first day of life | Applied to dorsal skin between 7 and 57 weeks |
|---|---|---|
| 1 | 1 mg. in 0·02 ml. 3% aqueous gelatin (a.g.) | 0·3 ml. of 0·15% MC in acetone twice weekly for 25 weeks, then 0·3 ml. of 0·3% MC in acetone twice weekly for 25 weeks (Groups 1–3) |
| 2 | 300 μg. MC „ „ | |
| 3 | 100 μg. MC „ „ | |
| 4 | 1 mg. „ „ „ | 0·3 ml. acetone only twice weekly for 50 weeks |
| 5 | 0·02 ml. a.g. | As for Groups 1–3 |

until weaning. Thereafter they were segregated by sex and treatment group. Topical applications to the skin were begun when they were 7 weeks old.

The results of the experiment are summarized in Tables II, III, and IV.

In females of Groups 1 to 3, which received one subcutaneous injection of MC at birth, followed by applications of MC to the skin, both the proportion of rats which developed sarcomas and the average induction-time for these tumours tended to vary directly with the initial dose of MC injected. In males, the incidence of sarcomas was similar in Groups 1 and 2 (1 mg. and 300 μg. MC at birth, respectively) but reduction of the dose of MC to 100 μg. (Group 3) decreased the incidence of sarcomas and increased the average time of their induction to levels comparable with those in females of the same group. In both sexes, neonatal injection of MC followed by topical applications of acetone (Group 4) was associated with a lower incidence of sarcomas than neonatal injection of MC followed by applications of MC in acetone (Group 1).

These results were analysed by the Gehan modification of the Wilcoxon rank test, a method which takes into account both incidence and induction time. The

TABLE II.—*Experiment I. Injection-site Sarcomas*

| Group | Number of rats alive at weaning | Treatment: S.c. at birth | Treatment: Applied to skin from 7th week | Number of rats which developed sarcomas at site of injection | Average age at death of sarcoma-bearing rats (weeks) |
|---|---|---|---|---|---|
| 1 | 17 ♂ | 1 mg. MC/a.g. | MC/acetone | 13 ♂ (76·5%) | 29·5 |
| | 15 ♀ | | | 12 ♀ (80·0%) | 32·8 |
| 2 | 18 ♂ | 300 μg. MC/a.g. | MC/acetone | 16 ♂ (88·9%) | 31·4 |
| | 9 ♀ | | | 5 ♀ (55·6%) | 33·8 |
| 3 | 10 ♂ | 100 μg. MC/a.g. | MC/acetone | 3 ♂ (30·0%) | 57·7 |
| | 12 ♀ | | | 3 ♀ (25%) | 43·3 |
| 4 | 9 ♂ | 1 mg./a.g. | Acetone only | 5 ♂ (55·6%) | 24·8 |
| | 12 ♀ | | | 5 ♀ (41·7%) | 33·0 |
| 5 | 18 ♂ | a.g. only | MC/acetone | 0 — | — |
| | 14 ♀ | | | 0 — | — |

TABLE III.—*Experiment I. Epithelial Skin Tumours*

| Group | Number of rats alive at weaning | Treatment: Injected at birth | Treatment: Applied to skin from seventh week | Number of rats which developed epithelial skin tumours | Average time of appearance of first skin tumours from start of application of MC to skin (weeks) | Times of death of rats which failed to develop epithelial skin tumours (weeks from first application of MC to skin) |
|---|---|---|---|---|---|---|
| 3 | 10 ♂ | 100 μg. MC/a.g. | MC/acetone | 10 ♂ (100%) | 31·0 | — |
| | 12 ♀ | | | 10 ♀ (83·3%) | 34·2 | 25, 30 |
| 5 | 18 ♂ | a.g. only | MC/acetone | 15 ♂ (83·3%) | 30·0 | 24, 28, 28 |
| | 14 ♀ | | | 14 ♀ (100%) | 30·4 | — |

TABLE IV—*Experiment I. Skin Tumour Incidence at Various Times After Start of Topical Treatment with MC*

| Group | Treatment at birth | Sex | 30 weeks %TBR* | 30 weeks AT/S† | 35 weeks %TBR | 35 weeks AT/S | 40 weeks %TBR | 40 weeks AT/S | 45 weeks %TBR | 45 weeks AT/S |
|---|---|---|---|---|---|---|---|---|---|---|
| 3 | 100 μg. MC/a.g. | ♂ | 50 | 0·7 | 90 | 2·5 | 100 | 8·0 | 100 | 11·9 |
| | | ♀ | 8·3 | 0·2 | 30 | 0·4 | 100 | 4·2 | 100 | 8·0 |
| 5 | a.g. only | ♂ | 20 | 0·3 | 87 | 3·4 | 100 | 5·4 | 100 | 10·0 |
| | | ♀ | 57 | 0·8 | 93 | 3·4 | 100 | 4·0 | 100 | 5·6 |

* %TBR = Percentage of survivors with skin tumours.
† AT/S = Average number of skin tumours per survivor.

outcome of this analysis is depicted in Fig. 1. The risk of sarcoma development was found to be significantly ($0{\cdot}05 > P > 0{\cdot}02$) less in females of Group 4 than in females of Group 1. The difference in risk was also significantly ($P < 0{\cdot}01$) less in the females of Group 2 than in the males of the same group. No other significant differences were found.

FIG. 1.—Experiment I—Distributions of time to appearance of sarcoma. Analysis by the Gehan modification of the Wilcoxon rank test. Each point on the curves was corrected to allow for deaths from intercurrent disease.

### *Epithelial skin tumours*

Sarcomas tended to arise earlier at the site of injection of MC than epithelial tumours induced by the application of MC to the skin. Such sarcomas grew rapidly so that animals had to be killed within 1 to 3 weeks of their first appearance. Thus, the development of sarcomas precluded proper analysis of the development of skin tumours in Groups 1, 2 and 4. The same problem did not arise in animals of Group 3 (100 $\mu$g. MC at birth followed by application of MC to skin) or in Group 5 (aqueous gelatin at birth followed by applications of MC to skin). Virtually 100 % of rats in these 2 groups developed epithelial tumours of the skin; the only animals which failed to do so died early in the experiment as a result either of sarcoma (2 female rats in Group 3) or of intercurrent disease (3 male rats in Group 5). The average time of appearance of the first skin tumour in individual animals was shorter in rats of Group 5 than those in Group 3, a difference which was more marked in females (Table III). This was analysed by the Gehan modification of the Wilcoxon rank test and also by the third Extreme Value Distribution method; both methods yielded similar results. Among males, there was no difference between Groups 3 and 5. In females, however, Group 5

animals developed first skin tumours significantly ($P < 0.01$) earlier than Group 3 animals.

The enhanced susceptibility of animals in Group 5 as compared with that of rats in Group 3 is reflected also in the multiplicity of skin tumours recorded at 35 weeks (Table IV). By the fortieth week, however, this difference had disappeared.

*Histological observations*

(a) *Injection-site tumours.*—Most of these neoplasms were poorly differentiated pleomorphic or spindle cell lesions, often with bizarre binucleate and multinucleate cells and regions of myxomatous degeneration, haemorrhage and necrosis. Metastatic deposits of sarcoma were seen in regional lymph nodes and/or lungs in 4 rats—3 from Group 1 and 1 from Group 4.

(b) *Epithelial skin tumours.*—A wide variety of epithelial neoplasms was encounted, comprising squamous carcinomas, mixed basi-squamous lesions and basal cell tumours. The detailed histological structure of these lesions will be described elsewhere, but the salient features in each group were as follows. *Squamous carcinomas* ranged from well-differentiated tumours to anaplastic forms growing as irregular columns of cells. Dermal invasion was variable in extent but the panniculus carnosus muscle was always infiltrated. *Basi-squamous tumours* were occasionally encountered. They were classified into 2 broad categories according to the predominating type of cell and, in most instances, a clear preponderance of basal or squamous elements was found. *Basal cell tumours* were commonly seen and were highly complex in structure. Two main types were distinguished—" pure" and " mixed " basal cell lesions. The " pure " basal cell tumours were composed of cells growing either as solid masses or in open reticular and " adenomatoid " patterns. The " mixed " basal cell tumours presented an elaborate histological picture with combinations of undifferentiated basal cells, sebaceous gland elements and hair follicles. Some lesions showed a wide variety of cell types; others presented a more homogenous appearance in which hair follicles or sebaceous glands predominated.

### *Experiment II: Effect of Neonatally Administered* 3,4-*benzopyrene* (*BP*) *on Response of Mice to Subsequent Topical Applications of BP*

Newborn mice were allocated to 4 groups for treatment as shown in Table V.

TABLE V.—*Experiment II. Details of Treatment*

| Group | Injected subcutaneously during first day of life | Applied to dorsal skin between 6 and 25 weeks |
|---|---|---|
| 1 | 40 μg. BP/0·02 ml. a.g. | 0·2 ml. 0·025% BP in acetone twice weekly |
| 2 | 10 μg. BP/0·02 ml. a.g. | 0·2 ml. 0·025% BP in acetone twice weekly |
| 3 | 0·02 ml. a.g. | 0·2 ml. 0·025% BP in acetone twice weekly |
| 4 | 40 μg. BP/0·02 ml. a.g. | 0·2 ml. acetone twice weekly |

All mice born on the same day were randomized, irrespective of the litter to which they belonged, between the 4 groups. After injection on the first day of life, they were distributed amongst the mothers such that each mother was given neonates from only 1 group. At weaning, the treated mice were segregated by group and sex. They were vaccinated against ectromelia when 4 weeks old and applications to the skin were begun when they were 6 weeks old. The results of the experiments are summarized in Fig. 2 and 3 and Table VI. No sarcomas developed at the site of neonatal injection in this experiment.

In the 3 groups which received topical applications of BP, the distributions of times of development of the first papilloma in individual mice were very similar (Fig. 2). However, if distributions of times of appearance of the first malignant

FIG. 2.—Experiment II—Distributions of time to appearance first papilloma in response to application of BP to the skin.

TABLE VI.—*Experiment II. Average Number of Papillomas and Carcinomas at Various Times*

| Group | Treatment at birth | Treatment from sixth to twenty-fifth week of age | Number of animals alive at 10 weeks (♂ + ♀) | Average number of papillomas and carcinomas per survivor at: 22 weeks | 26 weeks | 30 weeks | Average number of carcinomas per survivor at: 30 weeks | 36 weeks |
|---|---|---|---|---|---|---|---|---|
| 1 | 40 µg. BP/a.g. | BP | 34 | 0·2 | 1·6 | 4·1 | 0·1 | 0·8 |
| 2 | 10 µg. BP/a.g. | BP | 56 | 0·4 | 2·6 | 6·0 | 0·3 | 1·7 |
| 3 | 0·02 ml. a.g. | BP | 41 | 0·6 | 2·2 | 5·2 | 0·3 | 0·5 |
| 4 | 40 µg. BP | Acetone | 36 | — | — | — | — | — |

tumour are considered (Fig. 3), there is a significant delay ($P < 0{\cdot}025$, for method of analysis, see Grant, Roe and Pike, 1966) in the group which received 40 $\mu$g. BP at birth compared with those receiving 10 $\mu$g. BP or aqueous gelatin only.

Since these analyses only took account of the first papilloma (or carcinoma) in each mouse, a further analysis was made. By the use of the Wilcoxon rank test, the number of tumours (benign and malignant) in the survivors at 26 weeks and 30 weeks of age, in males and females separately, and in both sexes combined,

FIG. 3.—Experiment II—Distributions of time to appearance of first carcinoma in response to application of BP.

is shown in Table VII. In males and females there were fewer skin tumours in the group which received 40 $\mu$g. BP at birth than in the groups which received 10 $\mu$g. BP or aqueous gelatin only; these differences were statistically significant for females at 30 weeks in the 40 $\mu$g. BP/10 $\mu$g. BP comparison, for males plus females at 26 weeks in the 40 $\mu$g./10 $\mu$g. BP comparison and at 30 weeks for both 40 $\mu$g. BP/10 $\mu$g. BP and 40 $\mu$g. BP/aqueous gelatin comparisons. Analysis of malignant tumours by the Wilcoxon rank test also indicated a significantly reduced response in the 40 $\mu$g. BP-treated group.

TABLE VII.—*Experiment II*

Probability that differences between groups in average number of tumours per surviving mouse at different times after start of skin painting were due to chance (2-tailed significance levels *; a minus sign means that incidence in the first group is less than that in the second).

| Groups compared (designated by treatment 24 hours). | Total tumours, benign and malignant, Age 26 weeks: Males | Females | All | 30 weeks: Males | Females | All | Malignant tumours, Age 36 weeks: Males | Females | All |
|---|---|---|---|---|---|---|---|---|---|
| 40 μg. BP/10 μg. BP | −0·17 | −0·18 | *−0·04* | −0·31 | *−0·02* | *−0·01* | *−0·03* | *−0·03* | *−0·001* |
| 40 μg BP/a.g. | −0·26 | −0·19 | −0·18 | −0·19 | −0·07 | *−0·03* | *−0·01* | −0·44 | *−0·02* |
| 10 μg. BP/a.g. | +0·88 | +0·66 | +0·48 | −0·89 | +0·44 | +0·58 | −0·70 | +0·26 | +0·61 |

* For the purpose of this analysis animals in the two groups to be compared were ranked according to the number of skin tumours each bore at the stated time and the significance levels were calculated by the use of the Wilcoxon rank test.

Some tumours of other sites were observed; the most common were pulmonary adenomas which had a higher incidence in the groups receiving 40 μg. BP at birth irrespective of other treatment. Seven of the mice given BP at birth developed malignant lymphoma.

### *Histological observations*

The range of tumours encountered was narrower than in the rats of Experiment I. All those regarded as benign were squamous papillomas, some pedunculated, some sessile. Some of the sessile tumours showed compression of the dermis, and their general appearance suggested that they originated from hair follicles. All the tumours categorized as malignant were squamous carcinomas which showed active invasion of the panniculus carnosus muscle.

### *Experiment III: Effect of Neonatally Administered 3-Methylcholanthrene (MC) on Response of Mice to Subsequent Topical Applications of MC*

Newborn mice were allocated to 6 groups for treatment, as shown in Table VIII. The times at which mice in Groups 1–5 developed their first papillomas

TABLE VIII.—*Experiment III. Details of Treatment*

| Group | Injected subcutaneously during first 24 hours of life | Applied to dorsal skin between 6 and 20 weeks |
|---|---|---|
| 1 | 125 μg. MC/0·02 ml. Tricaprylin | 0·2 ml. 0·1% MC in acetone twice weekly (Groups 1–5) |
| 2 | 62 μg. ,, ,, ,, | |
| 3 | 31 μg. ,, ,, ,, | |
| 4 | 0·02 ml. Tricaprylin | |
| 5 | No injection | |
| 6 | 125 μg. MC/0·2 ml. Tricaprylin | 0·2 ml. acetone twice weekly |

and first carcinomas are depicted in Fig. 4 and 5. No skin tumours developed in Group 6. The appearance of first papillomas was slightly delayed in Groups 3 and 5, compared with other groups, but there was no difference in the rates at which Groups 1–5 developed first carcinomas. The total number of tumours (benign and malignant) in surviving animals (Table IX) was analysed by the Wilcoxon rank test (Table X). Statistically significant differences in both directions were seen between groups treated differently at birth but painted similarly with MC from the age of 6 weeks, and it was impossible to discern a meaningful pattern in the picture as a whole.

As shown in Fig. 6, sarcomas developed at the site of neonatal injection of MC in a number of animals, the incidence tending to vary directly with the initial dose of MC. The incidence of sarcomas was highest in the group injected with 125 μg. MC at birth which subsequently had MC applied to the skin.

FIG. 4.—Experiment III—Distributions of time to appearance of first papilloma in response to application of MC to the skin.

TABLE IX.—*Experiment III. Average Number of Papillomas and Carcinomas at Various Times*

| Group | Treatment at birth | Treatment from sixth to twentieth week of age | Number of animals alive at 10 weeks (♂ + ♀) | Average number of papillomas and carcinomas per survivor at: 16 weeks | 21 weeks | 26 weeks | Average number of carcinoma per survivors at: 26 weeks | 30 weeks |
|---|---|---|---|---|---|---|---|---|
| 1 | 125 μg. MC | MC | 40 | 0·5 | 5·0 | 8·9 | 0·4 | 1·2 |
| 2 | 62 μg. MC | MC | 43 | 0·4 | 3·3 | 8·8 | 0·4 | 1·2 |
| 3 | 31 μg. MC | MC | 43 | 0·2 | 2·0 | 6·9 | 0·6 | 0·9 |
| 4 | Tricaprylin | MC | 30 | 0·1 | 3·4 | 7·1 | 0·6 | 1·3 |
| 5 | None | MC | 40 | 0·1 | 1·8 | 6·5 | 0·5 | 1·3 |

FIG. 5.—Experiment III—Distribution of time to appearance of first carcinoma in response to application of MC to the skin.

TABLE X.—*Experiment III*

Probability that difference between groups in average number of tumours per surviving mouse at different times after start of skin painting were due to chance (2-tailed significance levels *; a minus sign means that incidence in the first group is less than that in the second.)

| Groups compared (designated by treatment 24 hrs) | Benign and Malignant Tumours 21 weeks Males | Females | All | 26 weeks Males | Females | All | Malignant Tumours 30 weeks Males | Females | All |
|---|---|---|---|---|---|---|---|---|---|
| 125 µg. MC/ Tricaprylin | +0·08 | +0·22 | +0·02 | +0·20 | +0·14 | +0·07 | −0·91 | −0·35 | −0·68 |
| 125 µg. MC/None | +*0·00006* | +*0·04* | +*0·0001* | +0·38 | +*0·0002* | +*0·008* | +0·25 | −*0·05* | −0·89 |
| 62 µg. MC/ Tricaprylin | −0·64 | +0·87 | −0·79 | +0·11 | +0·41 | +0·07 | −0·20 | +0·57 | −0·62 |
| 62 µg. MC/None | +*0·05* | +*0·05* | +*0·005* | +0·22 | +*0·02* | +*0·008* | −0·89 | −0·90 | −0·84 |
| 31 µg. MC/ Tricaprylin | −*0·03* | −0·18 | −*0·01* | +0·73 | −0·86 | +0·88 | −*0·01* | +0·99 | −0·10 |
| 31 µg. MC/None | −0·97 | +0·75 | +0·86 | −0·91 | +0·06 | +0·35 | −0·13 | −0·90 | −0·16 |
| Tricaprylin/None | +*0·01* | +0·90 | +*0·003* | −0·70 | +0·20 | +0·58 | +0·17 | −0·35 | +0·70 |

*See footnote to Table VII.

FIG. 6.—Experiment III—Distribution of time to appearance of first sarcoma in response to neonatal injection of MC.

The histological appearances of skin tumours were similar to those encountered in Experiment II.

Lung adenomas, usually multiple, arose in response to the neonatal injection of carcinogen. As in the first experiment with benzopyrene, their incidence varied with the dose of carcinogen given in the first 24 hours of life. Six cases of malignant lymphoma were encountered in the MC-treated mice but their incidence was not related to the dose of carcinogen given neonatally.

### DISCUSSION

In contrast to the findings reported by Anderson (1962), local sarcomas arose in rats injected with 1 mg. or 300 μg. MC at birth (Table II). The subsequent application of MC to the skin of rats injected with 1 mg. MC at birth significantly hastened the appearance of sarcomas (Fig. 1.) In mice, applications of MC to the skin enhanced the induction of sarcomas at the site of injection at birth of 125 μg. MC (Fig. 6). Prehn (1963) reported similar observations.

In Experiment I, 100 μg. MC injected at birth delayed by an average of 4 weeks as compared with solvent-injected controls, the appearance of epithelial skin tumours in female rats (Tables III and IV). This difference was significant ($P < 0{\cdot}01$). However, no difference of the same kind was seen in males and the effect of the delay had disappeared by the fortieth week (Table IV). It should also be stressed that the epithelial tumours induced by MC in Experiment I were of a variety of histological types, and that Anderson's reference to such lesions as " carcinomata " is equivocal.

No clear pattern emerged from the results of Experiment III in which mice were treated with various doses of MC at birth, and subsequently painted with the same carcinogen (Fig. 4 and 5 and Tables IX and X). It is possible in this case that the concentration of MC applied to the skin was too high and that a small difference in sensitivity in groups treated differently at birth was thereby obliterated.

Some support for Anderson's (1962) findings came from Experiment II: 40 $\mu$g. BP injected at birth significantly delayed the appearance of carcinomas (Fig. 3) and significantly reduced the average numbers of papillomas and carcinomas per survivor at various times during the experiment (Tables VI and VII). It is important to see whether this result can be confirmed.

The experiments described do not, unfortunately, succeed in their main purpose—namely to define the circumstances under which the phenomenon described by Anderson (1962) occurs. However, they do show that if the neonatal administration of carcinogens really does reduce the response of animals to subsequent exposure to the same carcinogen, the reduction is small and depends very much on the doses of the carcinogen given at birth and subsequently.

We feel that until these conditions have been better defined there is little point in trying to investigate the mechanism involved.

### SUMMARY

In rats, 3-methylcholanthrene (MC) applied to the skin from the age of 7 weeks significantly enhanced the induction of sarcomas at the site of the subcutaneous injection of 1 mg. MC on the first day of life.

In females, but not in males, the subcutaneous injection of 100 $\mu$g. MC in aqueous gelatin at birth delayed epithelial tumour-induction in response to the repeated application of MC to the skin from the seventh week onwards, as compared with rats injected with aqueous gelatin only at birth.

In mice, MC injected at birth at 3 different dose levels (125 $\mu$g., 62 $\mu$g. and 31 $\mu$g.) had no consistent effect on skin tumours arising in response to the subsequent cutaneous application of MC.

In mice, the injection of 40 $\mu$g. but not of 10 $\mu$g. 3,4-benzopyrene (BP) at birth reduced the number of skin tumours which arose in response to subsequent weekly applications of BP to the skin.

The results are discussed in the light of those reported by Anderson (1962).

This investigation has been supported by grants to the Chester Beatty Research Institute, Institute of Cancer Research: Royal Cancer Hospital, from the Medical Research Council and the British Empire Cancer Campaign for Research, and by the Public Health Service Grant No. CA–03188–11 from the National Cancer Institute, U.S. Public Health Service.

### REFERENCES

ANDERSON, M. R.—(1962) *Nature, Lond.*, **194**, 1290.

BILLINGHAM, R. E. AND BRENT, L.—(1959) *Phil. Trans. R. Soc.* (B) **242**, 439.

BILLINGHAM, R. E., BROWN, J. B., DEFENDI, V., SILVERS, W. K. AND STEINMULLER, D.—(1960) *Ann. N.Y. Acad. Sci.*, **87**, 457.

GRANT, G., ROE, F. J. C., AND PIKE, M. C.—(1966) *Nature, Lond.*, **210**, 603.
GREEN, H. N.—(1954) *Br. med. J.*, ii, 1374.
KELLY, M. G. AND O'GARA, R. W.—(1961) *J. natn. Cancer Inst.*, **26**, 651.
O'GARA, R. W. AND KELLY, M. G.—(1963) *Proc. Am. Ass. Cancer Res.*, **4**, 49.
PREHN, R. T.—(1963) *J. natn. Cancer Inst.*, **31**, 791.
ROE, F. J. C., ROWSON, K. E. K. AND SALAMAN, M. H.—(1961) *Br. J. Cancer*, **15**, 515.
WALTERS, M. A.—(1966) *Br. J. Cancer*, **20**, 148.